# System-Specific Complex Interactions Shape Soil Organic Carbon Distribution in Coastal Salt Marshes

**DOI:** 10.3390/ijerph17062037

**Published:** 2020-03-19

**Authors:** Dan Yang, Xin-Yu Miao, Bo Wang, Ren-Ping Jiang, Teng Wen, Mao-Song Liu, Cheng Huang, Chi Xu

**Affiliations:** 1 School of Life Sciences, Nanjing University, Nanjing 210023, China; mg1730080@smail.nju.edu.cn (D.Y.); miaoxy@smail.nju.edu.cn (X.-Y.M.); bowang@smail.nju.edu.cn (B.W.); jiangrp@smail.nju.edu.cn (R.-P.J.); huangcheng@nju.edu.cn (C.H.); 2 School of Geography Sciences, Nanjing Normal University, Nanjing 210023, China; wenteng@njnu.edu.cn

**Keywords:** carbon cycle, soil organic carbon, soil properties, structural equation model, trophic interaction, wetland

## Abstract

Coastal wetlands provide many critical ecosystem services including carbon storage. Soil organic carbon (SOC) is the most important component of carbon stock in coastal salt marshes. However, there are large uncertainties when estimating SOC stock in coastal salt marshes at large spatial scales. So far, information on the spatial heterogeneity of SOC distribution and determinants remains limited. Moreover, the role of complex ecological interactions in shaping SOC distribution is poorly understood. Here, we report detailed field surveys on plant, soil and crab burrowing activities in two inter-tidal salt marsh sites with similar habitat conditions in Eastern China. Our between-site comparison revealed slight differences in SOC storage and a similar vertical SOC distribution pattern across soil depths of 0–60 cm. Between the two study sites, we found substantially different effects of biotic and abiotic factors on SOC distribution. Complex interactions involving indirect effects between soil, plants and macrobenthos (crabs) may influence SOC distribution at a landscape scale. Marked differences in the SOC determinants between the study sites indicate that the underlying driving mechanisms of SOC distribution are strongly system-specific. Future work taking into account complex interactions and spatial heterogeneity is needed for better estimating of blue carbon stock and dynamics.

## 1. Introduction

Coastal areas account for only 4% of the Earth’s land surfaces, yet accommodate over one third of the world’s total population [1]. Coastal wetlands are among the most important ecosystems, providing a wide range of critical ecosystem services to human well-being, including climate mitigation, flood control, shoreline stabilization, storm protection, sediment and nutrient retention, fishery production and biodiversity conservation [2,3]. In recent years, there has been increasing recognition that coastal wetlands, as a long-term carbon sink (referred to as a major component of “blue carbon”), play a key role in climate change mitigation [4]. Previous studies have shown that many coastal wetland ecosystems, such as salt marshes, mangroves and seagrass beds, are storing a considerable amount of carbon with high densities. These coastal ecosystems only account for 0.07%–0.22% of the Earth’s Land surfaces, but can capture up to 0.08–0.22 Pg carbon every year [5]. Particular interest has been given to coastal salt marshes because they have shown a surprisingly high capability of carbon sequestration, as reflected by an average rate of around 245 g C·m^−2^·per year, approximately 40 times of that of temperate forest ecosystems [6,7], making coastal salt marshes one of the most important carbon pools at regional and global scales. 

Soil organic carbon (SOC) represents a major proportion of the carbon pool in coastal salt marshes as well as in many other ecosystems [2]. SOC concentration in salt marshes can serve as a useful indicator of climate change [8]. In the meantime, changes in SOC storage in salt marshes are expect to produce profound impacts on the global carbon cycle [9]. However, so far, accurate information on SOC distribution in coastal salt marshes remains limited, resulting in uncertainties when estimating SOC stock, especially at broad spatial scales [10,11,12,13]. SOC stock is determined by many factors, ranging from climate conditions at a macro scale to soil properties and microbial activities at a micro scale [14,15]. Extensive research effort has been devoted to mapping SOC distributions across a range of spatial scales and to elucidating the determinants in coastal salt marshes [13,16,17,18], as they are essential to understanding and predicting the dynamics of blue carbon. Estimation of SOC distribution at landscape and regional scales has been strongly dependent on spatial extrapolation from site-level data and/or ecosystem process modelling, whose accuracy also relies on validation based on field data [13,18]. While information from field observations is critical, sampling points of field data are often sparse, largely restricted by the labor-intensive nature of SOC measurements. For the sampling design of SOC surveys, a generally adopted approach is that much of the resource (sampling density) is allocated to account for the difference between distinct habitat or ecosystem types, while only very few sampling points are allocated between similar systems (in terms of habitat conditions, vegetation type, species composition and structure, etc.) based on the assumption that such similar systems would have slight differences. However, it is unclear if, and under what conditions, this important assumption holds. Previous work has suggested that even between seemingly homogeneous habitat conditions, horizontal and vertical distributions of SOC, as well as of their driving mechanisms, could vary to a substantial degree [16]. When it comes to coastal salt marshes that are strongly subject to complex hydrological and sedimentation processes, it has been documented that SOC distribution had significant spatial variations between different systems with similar habitat conditions [16]. However, information on between-site differences in SOC distributions and their driving factors remains lacking. More importantly, complex ecological interactions, especially indirect effects and trophic interactions, have been increasingly demonstrated to play important roles in many aspects (e.g., species co-existence, spatial patterning, non-linear vegetation dynamics, ecosystem functioning and resilience, etc.) of salt marsh ecosystems as well as other ecosystem types [19,20,21,22,23,24,25,26,27]. Yet, it remains poorly understood if and to what extent such between-site SOC differences can be attributed to those complex ecological interactions, as reflected by the lack of consideration of those interactions in a majority of existing studies on the estimation of SOC stock. This knowledge gap leaves important uncertainties for estimating blue carbon stock and dynamics in the face of rapid changes in the climate and in anthropogenic activities. 

To address this gap, in this study, we conducted detailed field investigations to compare SOC distribution and determinants between two salt marsh sites along the Yellow Sea coast of Eastern China. We selected two representative inter-tidal mudflat sites in the core area of two national nature reserves, respectively. These sites, with a spatial distance of 65 km, have similar ecosystem properties in terms of climate, tidal location, vegetation type, species composition, and anthropogenic activities, making them a suitable natural experimental system for our study. Here, we focus on the horizontal and vertical distributions of SOC at a landscape scale. We used regression analysis to examine the effects of a set of biotic and abiotic environmental factors on SOC distribution. To quantify the role of complex ecological interactions, we used structural equation modeling to infer the direct and indirect effects of those factors on SOC distribution. We expect to provide a better understanding of carbon cycle in costal salt marshes, with useful implications for more accurate estimations of blue carbon at landscape scales.

## 2. Materials and Methods 

### 2.1. Study Area

**Figure 1:**
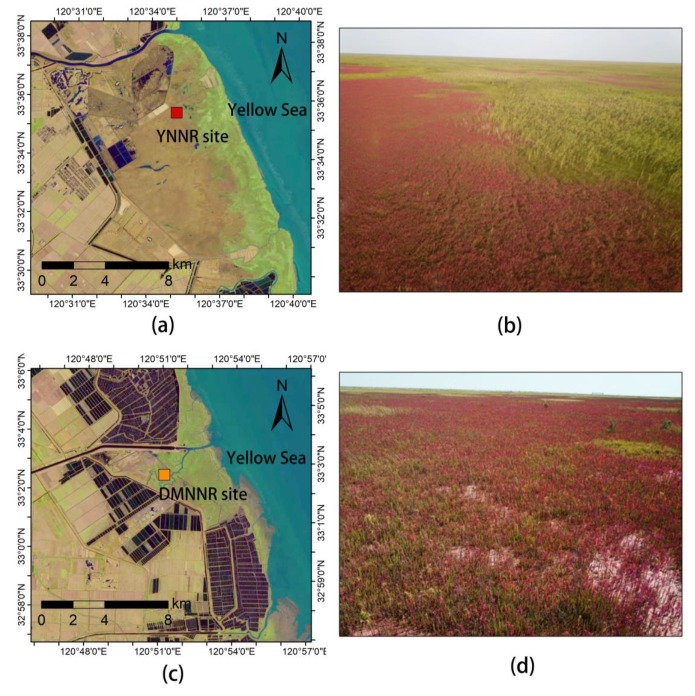
Locations and photos of the study sites. Left column: Landsat 8 OLI remotely sensed image acquired on December 5, 2017; right column: drone photos taken above the study sites in October, 2018, showing the *Suaeda salsa* vegetation patches in red color (photo credit: Xin-Yu Miao). (**a**) Landsat image of the YNNR site, (**b**) drone photo of the YNNR site, (**c**) Landsat image of the DMNNR site, (**d**) drone photo of the DMNNR site.

Our two study sites are located in the core areas of the Yancheng National Nature Reserve (YNNR, 120.58°E, 33.59°N) and Dafeng Milu National Nature Reserve (DMNNR, 120.84°E, 33.04°N), respectively (Figure 1). This region has a mean annual temperature of 13.7–14.6 °C and a mean annual precipitation of 980–1070 mm [28]. Irregular tidal flooding occurs twice a day. The YNNR was established in 1983, aiming at protecting red-crown cranes (*Grus japonensis*) and their habitats. The DMNNR was established in 1986, aiming at protecting Père David’s Deer (*Elaphurus davidianus*, Milu in Chinese pinyin) and their habitats. Both nature reserves are now under administration at the national level (the highest level in the Chinese nature reserve system) with implementation of strict protection. Human activities are strictly forbidden in the core areas of the reserves. 

These two nature reserves accommodate the largest and most intact natural inter-tidal mudflat ecosystems in China. Distinct vegetation types with clear boundaries are present along the spatial gradient from the sea to the land. The pioneer species *Spartina alterniflora* dominates the low-tidal zone, while reeds (*Phragmites australis*) are the dominant vegetation (with other plant species such as *Aeluropus sinensis*, *Imperata cylindrical*, and *Scripus karuizawensis* in presence) in the high-tidal zone [29]. In between, *Suaeda salsa*, as the dominant species [29], forms spatially extensive vegetation patches (see the photos in Figure 1). 

### 2.2. Soil Analyses 

We set up one study site in the YNNR and one in the DMNNR. Both sites are located in the *S. salsa* marshes, and present homogeneous habitat conditions, with a similar elevation of 7 m above the sea level and similar tidal conditions. We conducted field sampling during the peak growing season (July-September) in 2018. In each site, we randomly selected 30 quadrats sized 1 × 1 m^2^ with nearest-neighboring distances of 2–10 m. Within each quadrat, we collected soil samples at the depths of 0–10 cm, 10–30 cm and 30–60 cm. At each depth, three subsoil samples of 200 g were collected, bagged and stored within ice boxes. These three subsoil samples were then fully mixed, passed through 2-mm sieves and dried. For the measurement of SOC, they were grinded and passed through a 100-mesh sieve. Aboveground biomass (AGB) in each quadrat was collected, bagged and dried for 12 h to a constant weight in an 80 °C oven. In addition, previous studies have suggested that crabs can play an important role as ecosystem engineers, with important effects on many aspects of salt marsh ecosystems including plant performance and SOC accumulation [20,30,31]. Considering that spatial patterns can serve as useful indicators for inferring ecological process in many cases [24,26,32], we characterized local spatial patterns (within the quadrats) of crab (*Chiromantes dehaani* was the dominant species in our study sites) burrows to quantify the effects of crab activities in a comprehensive and simple way. To this end, we used two variables including crab burrowing density (BUD, measured by burrow number per m^2^) and mean distance of nearest neighboring burrows (MDNN, as an indicator of spatial pattern in terms of scatter vs. clump).

While SOC concentration could be influenced by numerous soil factors, it is only feasible to measure a limited set of soil variables in most field work due to the restriction of cost [33]. Here, we aimed to assess if and how the most fundamental soil properties can shape SOC distribution at a landscape scale. We therefore focused on the most commonly used variables that are readily available in almost all soil surveys, including bulk density (BD), pH, and electrical conductivity (EC, an indicator of saline condition). We did not include soil nutrient factors (such as N and P) in the subsequent statistical modeling, because they are often not readily available in all soil survey datasets and usually have highly system-specific relationships with soil carbon. Nor did we include soil moisture, because it is strongly dependent on fast-changing hydrological (tidal) conditions. We used standard methods to measure the soil variables following the protocols in [34]. 

### 2.3. Statistical Analyses 

As a first step, we compared SOC density as well as the biotic and abiotic habitat factors between the YNNR site and the DMNNR site. We then conducted multiple ordinary least square regression analysis to assess if these factors have direct effects on SOC distribution. We used the adjusted *R*^2^ of the full models to assess the predictive power of the selected variables for SOC distribution. We did not use stepwise-like model selection to avoid potential bias [35]. To further assess if indirect effects could possibly shape the SOC distribution, we used structural equation modeling to take into account the indirect effects of abiotic soil variables and crab burrows. Based on previous studies [20,30,31,36], we tested for the indirect effects of soil properties (BD, pH and EC) on SOC through affecting plant performance (AGB) and crab burrowing, and the indirect effects of crab burrowing on SOC through affecting plant AGB. These effects were represented by the following three specific causal pathways, (1) soil properties → plant AGB → SOC, (2) soil bulk density (hardness for burrowing) → crab burrowing → SOC and (3) soil bulk density → crab burrowing → plant AGB → SOC. We assumed that plant AGB and SOC may affect each other (forming a feedback). The explanatory variables showed a low VIF (variance inflation factor) value < 5, indicating a low level of multi-collinearity. All statistical analyses were conducted with R 3.3.1 [37] with the *piecewiseSEM* package [38] for structural equation modeling. 

## 3. Results

### 3.1. Between-Site Difference in SOC Distribution

**Figure 2:**
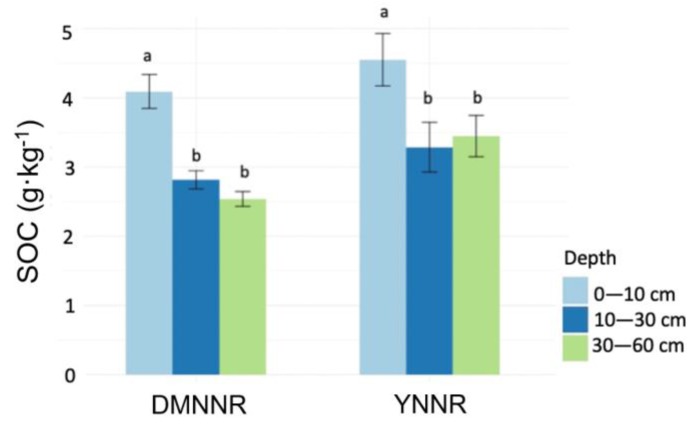
SOC densities (unit: g·kg^−1^, mean ± standard error) at the three soil layers of 0–10 cm, 10–30 cm and 30–60 cm in the two study sites. Different letters (a vs. b) above the error bars indicate significant differences (two-way ANOVA). *n* = 30.

Our results from the field investigation on 60 sampling quadrats showed that SOC density was 3.76 ± 1.26 g·kg^−1^ in the YNNR site and 3.15 ± 0.69 g·kg^−1^ (mean ± std) in the DMNNR site. The difference in the mean SOC density was less than 20%, but statistically significant (*t* test, *p* = 0.023). For all three soil layers, the DMNNR site presented slightly lower (but non-significant) SOC densities than the YNNR site (Figure 2). Both sites showed a similar vertical distribution pattern of SOC density, characterized by a higher density at the topsoil layer (0–10 cm) and lower densities at the subsoil layers (10–30 cm and 30–60 cm). 

**Figure 3:**
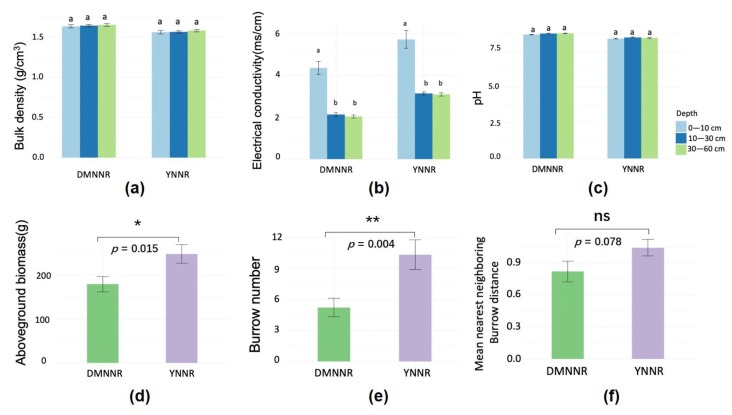
Soil properties (**a**–**c**), plant aboveground biomass (**d**), crab burrow number (**e**) and mean distance of nearest neighboring burrows (**f**) in the two study sites (mean ± standard error). In the upper panels, different letters (a vs. b) above the error bars indicate significant differences (two-way ANOVA). *: *p* < 0.05; **: *p* < 0.01; ns: *p* > 0.05; *n* = 30.

We also observed significant differences in the abiotic and biotic environmental factors between the two study sites (Figure 3). For example, the YNNR site presented higher EC, AGB and crab burrow numbers, but lower BD and pH than the DMNNR site. In addition, some factors such as EC showed a consistent vertical distribution pattern with SOC density. We then conducted statistical analyses to quantify the relationships between SOC density and the environmental factors.

### 3.2. Multiple Regression Analyses 

While the visual between-site comparison may provide an impression that the between-site similarities and differences of the environmental factors coincide with the observed SOC distribution patterns, the result from the multiple regression analyses suggests that they generally had weak power (indicated by the adjusted *R*^2^ of the full model) in explaining the SOC distribution, especially in the YNNR site. Importantly, the total explanatory power and the effects of the individual environmental factors (as explanatory variables) strongly varied between the two study sites. In the DMNNR site, BD and EC were significantly correlated with SOC at the 30–60 cm soil layer, and BUD was significantly correlated with SOC at the 0–10 cm layer. In contrast, only EC and MDNN had marginally significant correlations with SOC (*p* < 0.1) in the YNNR site. 

In short, the multiple regression models suggested that soil properties and crab burrowing could have significant effects on SOC distribution, but their effects were highly site-specific. Surprisingly, we did not find a significant effect of plant performance in terms of aboveground biomass in either study site.

### 3.3. Inferring Complex Interactions from Structural Equation Modeling

**Figure 4:**
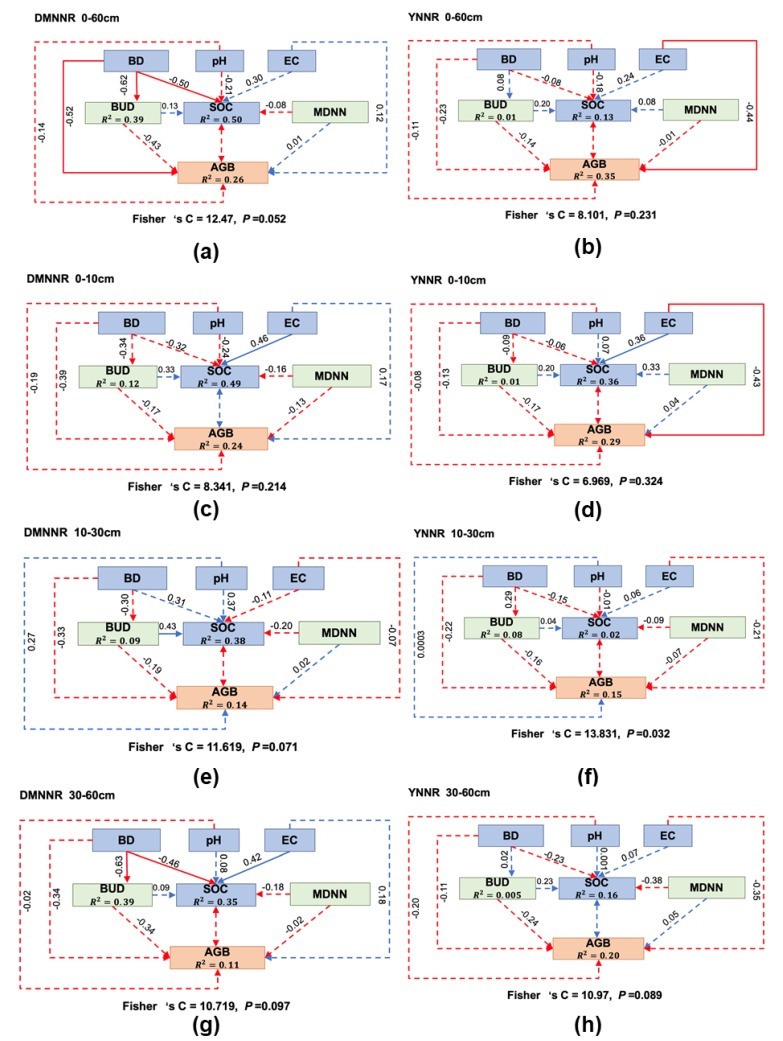
Results from structural equation models assuming complex interactions between soil, plants and crab activities. Solid arrows: *p* < 0.05; dashed arrows: *p* > 0.05; blue arrows: positive effects; red arrows: negative effects; *n* = 30.

Considering that indirect effects cannot be explicitly taken into account in multiple regression models, we constructed structural equation models to test for the indirect effects of soil properties and crab burrowing on SOC distribution. The results from the structural equation models showed that these indirect effects were indeed possibly at play (all models have overall *p* value lager than 0.05, suggesting that the causal pathways were possibly present, Figure 4). Looking at all soil layers as a whole, the direct and indirect effects can jointly explain 50% (in the DMNNR site) and 13% (in the YNNR site) of the observed variance in SOC distribution. The results suggested that taking into account the indirect effects can substantially increase the explanatory power, compared with regression models that only consider the direct effects. 

An important finding is that the explanatory power of the structural equation models substantially varied across different soil layers and between different study sites. There was a consistent pattern where the topsoil layer of 0–10 cm had the greatest explanatory power. However, closer scrutiny of the different soil layers between the two study sites reveal marked differences in the potential pathways of the environmental factors shaping SOC distribution. For example, the effect of the soil physical property (BD) was much more pronounced in the DMNNR site, while soil salinity (EC) presented a pronounced effect in the YNNR site. The two variables representing crab activities generally had weak effects on SOC density and plant performance (AGB). 

In brief, the assumed complex interactions were indeed possible for shaping SOC distribution, but they displayed remarkable differences between the two study sites.

## 4. Discussion

In this study, we conducted a detailed comparison between two inter-tidal sites to assess if and to what extent landscape-scale SOC distribution, as well as its driving mechanisms, can vary between natural salt marsh ecosystems with similar habitat conditions. Between the two study sites, we found an overall difference in SOC density of <20% (all soil layers in combination) and a similar vertical SOC distribution pattern (i.e., higher densities in topsoil and lower densities in subsoil). Despite these similarities, we found markedly different effects of biotic and abiotic environmental factors with complex interactions, suggesting that the driving mechanism of SOC distribution was strongly system-specific. 

Our finding on the vertical distribution pattern of SOC density agrees with many previous studies showing that SOC distribution tends to exhibit a decreasing trend towards deeper soil layers [17,18]. This pattern was robust across the two study sites with a spatial distance of 65 km. This result is in line with the view that, under natural conditions, aboveground plant residuals as the source of SOC input play a major role in driving this pattern. While this simple vertical distribution pattern makes intuitive sense, the potential underlying mechanisms and determinants are complex, as various factors could influence plant growth and decomposition rate, and in turn influence SOC density. This raises an important question: does this complexity hamper our ability to predict and estimate SOC distribution at large spatial scales? It has been suggested that it is indeed feasible to estimate SOC distribution using a set of common soil variables, including pH, salinity and soil texture, as they can well explain the spatial variance of SOC distribution in particular systems [17]. However, our work demonstrated that the relationships between the environmental factors and SOC density can be strongly system-specific, and sometimes can be rather weak. This finding provides an important caveat for extrapolating SOC density to a large scale from soil properties alone. 

Probably the foremost finding of this work is that complex interactions between soil, plants and crabs involving indirect effects can jointly shape SOC distributions in the investigated study systems. Our modeling results suggest that the consideration of indirect effects can improve model performance for predicting SOC distribution by up to 16% in terms of explanatory power (for instance, for the 10–30 cm soil layer in the DMNNR site, the multiple regression model had *R*^2^ of 0.22, (Table 1), whereas the structural equation model had *R*^2^ of 0.38 (Figure 4e)). The importance of such indirect effects, particularly those operating through trophic interactions, has been well documented in coastal salt marshes as well as in many other ecosystems [20,21,27,39]. For example, it has been documented that crabs can forage for fallen leaf litter and relocate this source of soil organic carbon to deeper burrow chambers [40]. This process can significantly alter SOC stock and increase the spatial heterogeneity of SOC distribution. However, so far, few attempts have incorporated indirect effects when it comes to the estimation of SOC distribution and dynamics. Our work suggests that burrowing density, reflecting the intensity of crab activities, can produce significant effects on SOC density in some cases (Table 1). One could expect that the micro-scale spatial pattern of burrows would influence SOC density as well, in the sense that clumped burrowing (corresponding with lower MDNN) might lead to strong intraspecific competition between crabs, thus reducing relocation of plant litter and SOC. However, this expectation is not supported by our data, as the observed effect of MDNN seems quite weak and elusive. One possibility is that the simple spatial patterning variable used in this study may be not sufficient to capture the effect. It is also possible that burrowing depth (not measured in the field) can act as a confounding factor, because it determines the vertical relocation of SOC. A better understanding of the crab effects requires further studies based on thoughtful experiments on burrowing activities. 

An important caveat should be noted when it comes to the interpretation of the model results. In our study, as well as in a majority of existing relevant studies (e.g., [15,17]), the measured soil properties and plant biomass only represent static situations. In a relative sense, SOC stock is a slow variable, driven by the long-term dynamics of soil and biotic variables, some of which are fast variables (e.g., soil salinity and crab burrowing activities). This long-standing problem of temporal scale mismatch may lead to weak correlations between SOC density and biotic and abiotic environmental variables, making it difficult to unravel the underlying mechanisms. In recent years, the development of ‘Internet of Things Technology’, combined with automatic monitoring equipment for environmental monitoring, has given rise to rapidly increasing high-resolution time-series data on ecosystem dynamics. These emerging technologies and big data combined with newly developed mathematical and modeling tools (e.g., Bayesian-network-based causal inference methods [41]) are expected to largely overcome the problems of data sparsity and scale mismatch.

Taken together, our study paves the way towards disentangling the complex interactions shaping SOC distribution in coastal salt marshes. It points to the necessity of incorporating these indirect effects for better understanding the mechanisms underlying carbon stock in coastal ecosystems. It also calls attention to spatial heterogeneity and system specificity for estimating blue carbon stock and dynamics.

## 5. Conclusions

By comparing between two inter-tidal salt marsh sites with similar habitat conditions in Eastern China, we observed minor differences in SOC storage and a similar vertical SOC distribution pattern across the soil depths of 0–60 cm. Despite these similarities, we found strongly different effects of biotic and abiotic environmental factors on SOC density distribution between the two study sites. Complex interactions involving indirect effects between soil, plants and macro-benthos (crabs) can provide important additional explanatory power to the models explaining SOC distribution, suggesting that these interactions may underpin SOC distribution at a landscape scale. Marked differences in the SOC determinants between the study sites indicate that the underlying driving mechanisms of SOC distribution are strongly system-specific. Future work, taking into account spatial heterogeneity and system specificity, is needed for improving the accuracy of estimates of blue carbon stock and dynamics.

## Figures and Tables

**Table 1:** Results from multiple regression models explaining soil organic carbon distributions.

Site	DMNNR			YNNR		
Soil Depth	0–10 cm	10–30 cm	30–60 cm	0–10 cm	10–30 cm	30–60 cm
**BD**	−0.23 ± 0.18	0.29 ± 0.21	−0.51 ± 0.23 *	−0.06 ± 0.19	−0.01 ± 0.25	−0.21 ± 0.20
**pH**	−0.20 ± 0.16	−0.11 ± 0.20	0.08 ± 0.20	0.07 ± 0.19	0.02 ± 0.26	0.09 ± 0.25
**EC**	0.43 ± 0.16 *	0.40 ± 0.20 ^†^	0.45 ± 0.20 *	0.35 ± 0.19 ^†^	−0.12 ± 0.23	0.17 ± 0.27
**AGB**	0.22 ± 0.16	−0.06 ± 0.18	−0.16 ± 0.17	−0.02 ± 0.20	−0.16 ± 0.22	0.07 ± 0.22
**BUD**	0.37 ± 0.17 *	0.42 ± 0.20 *	0.03 ± 0.25	0.20 ± 0.18	−0.03 ± 0.24	0.23 ± 0.22
**MDNN**	−0.14 ± 0.16	−0.20 ± 0.20	−0.18 ± 0.19	0.33 ± 0.18 ^†^	−0.12 ± 0.26	−0.44 ± 0.25 ^†^
**Adjusted** $R^{2}$	0.40	0.22	0.21	0.19	0.00	0.00

Standardized coefficients ± std are shown for the explanatory variables; ^†^ *p* < 0.1; * *p* < 0.05; BD: bulk density; EC: electrical conductivity; AGB: plant aboveground biomass; BUD: burrow density; MDNN: mean distance of nearest neighboring burrows; *n* = 30.
